# Error mapping controller: a closed loop neuroprosthesis controlled by artificial neural networks

**DOI:** 10.1186/1743-0003-3-25

**Published:** 2006-10-09

**Authors:** Alessandra Pedrocchi, Simona Ferrante, Elena De Momi, Giancarlo Ferrigno

**Affiliations:** 1 Nitlab, Bioengineering Department, Politecnico di Milano, Milano, Italy

## Abstract

**Background:**

The design of an optimal neuroprostheses controller and its clinical use presents several challenges. First, the physiological system is characterized by highly inter-subjects varying properties and also by non stationary behaviour with time, due to conditioning level and fatigue. Secondly, the easiness to use in routine clinical practice requires experienced operators. Therefore, feedback controllers, avoiding long setting procedures, are required.

**Methods:**

The error mapping controller (EMC) here proposed uses artificial neural networks (ANNs) both for the design of an inverse model and of a feedback controller. A neuromuscular model is used to validate the performance of the controllers in simulations. The EMC performance is compared to a Proportional Integral Derivative (PID) included in an anti wind-up scheme (called PIDAW) and to a controller with an ANN as inverse model and a PID in the feedback loop (NEUROPID). In addition tests on the EMC robustness in response to variations of the Plant parameters and to mechanical disturbances are carried out.

**Results:**

The EMC shows improvements with respect to the other controllers in tracking accuracy, capability to prolong exercise managing fatigue, robustness to parameter variations and resistance to mechanical disturbances.

**Conclusion:**

Different from the other controllers, the EMC is capable of balancing between tracking accuracy and mapping of fatigue during the exercise. In this way, it avoids overstressing muscles and allows a considerable prolongation of the movement. The collection of the training sets does not require any particular experimental setting and can be introduced in routine clinical practice.

## Background

Nowadays neuromuscular electrical stimulation allows simple clinical practice of rehabilitation therapy, even if some of its initial promises have failed. Indeed, the complex motor control performed by the Central Nervous System (CNS) is hard to reproduce by any artificial controller, even to recover a single function like gait, sit to stand or grasping.

Several studies were presented in the last years aiming at controlling such motor tasks by stimulation [1-4] and some commercial products are available in the market [5-8]. Functional stimulation allows conditioning muscular tone, reducing joint stiffness, increasing peripheral vascularisation, preventing ulcers and providing a good cardiorespiratory training. In addition functional neuromuscular stimulation provides the CNS with a complete afference of the motor function to be re-learnt offering promising advantages in the rehabilitation of incomplete spinal cord injured, stroke and ataxia patients [9,10].

In this frame, the development of sophisticated control systems is a crucial point in the design of neuroprostheses. Namely, the control should be able to let the limb track accurately the desired movement and to repeat the exercise as long as possible, even if fatigue occurs. The problem of fatigue is actually particularly amplified for artificial contraction because muscular fibres are activated synchronously, at higher frequency and in the opposite order with respect to the natural contraction.

A neuroprosthesis should be specifically calibrated on a single subject and even on a single session of each subject. The design has to face the well-known difficulties of controlling the human neuromuscular apparatus: non linear, time varying, redundant and very difficult to model analytically. In addition to these typical bioengineering problems, there is another crucial aspect in the design of a neuroprosthesis, i.e., making it easy to use in clinics. The real widespread use in clinical practice as well as the probability of being accepted by many patients strongly depend on short preparation and on exercise procedures being easy.

Most controllers available for functional neuroprostheses in clinical practice are feedforward (FF) [11-13]. They predefined a fixed stimulation pattern during the motor task. By definition, a FF controller did not include any correction on the basis of the current performance, limiting the possibility to track the time variability of the neuromuscular apparatus. On the contrary, several feedback (FB) controllers were proposed. Adaptive controllers [14] and PID controllers were designed for the purpose [15]: Veltink showed that the good tracking performance of PID controllers was offset by a considerable time lag between reference and actual joint angle, which became more marked when exercises were protracted in time. In order to reduce the time lag and to give the PID a FF guess, model-based controllers were combined with PID [1]. These included a neuro-musculo-skeletal model of the system to be controlled. Unfortunately, the large quantity of parameters required for the identification of the system to be controlled was difficult to be experimentally determined and, anyway, a long preparation for each patient was needed in each session. An attempt to reduce this problem was the replacement of the physiological model with a non-linear black-box model, such as an artificial neural network (ANNs). Chang et al. [16] proposed a NEUROPID controller composed by a neural network trained to behave as inverse model in the FF line and a fixed-parameter PID feedback controller, thereby making adjustments for residual errors, due to external disturbances, or to erroneous model identification. Results demonstrated an improvement of tracking performance with respect to Veltink [15], especially because of the reduction of the PID time lag. However, such controller still required long preparation for the PID setting and when fatigue increased, the controller was overstressing the stimulation inducing itself a very fast fatigue increase.

Abbas et al. [17-19] proposed a control system which used a combination of adaptive FF and FB control techniques. The FF adaptive controller was a pattern generator/pattern shaper (PG/PS), in which PG generated a stable oscillatory rhythm while PS (a single-layer neural network) took its input from PG and provided the muscles with stimulation. A fixed-parameter proportional-derivative (PD) FB controller enhanced disturbance resistance and supplemented the action of the FF controller. This controller showed a good performance both in simulation and in experimental sessions, with a good capability of controlling different subjects. The adaptive controller was demonstrated only to repeat one-pattern sequences. However, no particular evidences were reported by the authors about the efficacy of the controller in tracking fatigue. Even if it could be used with many patterns, this could strongly decrease the efficiency and velocity of the adaptive controller, being the architecture of PS multiplied by the number of patterns. In the study proposed by Jezernik et al. [20], a sliding mode controller was developed and demonstrated a good stability and robustness to parameter variations in an early stage of the movement, before the occurrence of fatigue. As discussed by the authors themselves, one of the main drawbacks of the controller is the time required for the tuning phase of the great number of parameters.

In a previous study developed by our research group [21], an adaptive control system (NEURADAPT) based on ANNs was designed to control the knee joint angle in accordance with desired trajectories, by stimulating quadriceps muscles. This strategy included an inverse neural model of the stimulated limb in the FF line and a ANN trained on-line to learn a PID behaviour in the feedback loop. Despite the encouraging results, the ANN in the feedback loop still relied on a PID: it needed the PID parameters identification phase and it also produced a considerable time lag between the reference and actual joint angle, due to the intrinsic delay of the integrative part of the PID function.

With the presented literature and these previous results as a starting point, the control strategy developed and presented in this study is totally free of a PID controller.

In order to combine the engineering requirements along with the clinical specifications, we designed a control system for a neuroprosthesis, called Error Mapping Controller (EMC), for a simple motor task such as knee flexion and extension. This neuroprosthesis was completely designed to identify the controller in the normal steps of clinical use of electrical stimulation, avoiding extra complex protocol procedures to the therapist and the patient.

## Methods

**Figure 1:**
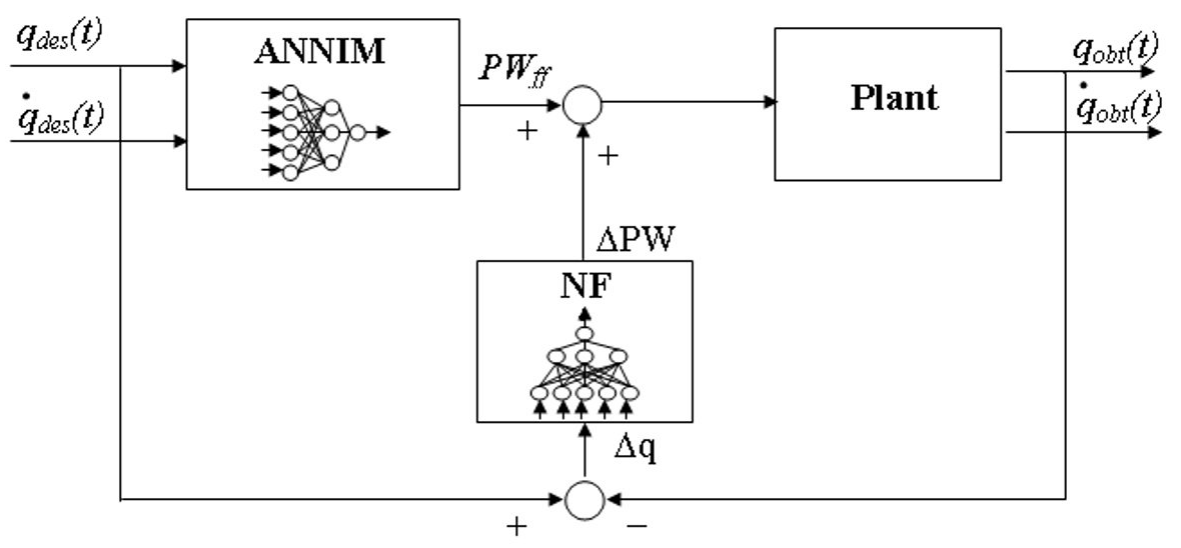
**EMC controller**. EMC structure.

EMC structure is reported in Figure 1. It included a FF ANN inverse model (ANNIM) of the system to be controlled and a neural network trained to compensate the fatigue effects in the FB loop, Neuro Feedback (NF). ANNIM stored a stable scheme of the motor apparatus and it was able to convert the planned desired movement (trajectory) into motor commands (pulse width of the stimuli). FB controller (NF) provided the correction of the motor command depending on the current error of the executed movement and on the estimation of the current fatigue level.

### Neuro-muscular skeletal model

In order to simulate neuromuscular skeletal features of the lower limb of a paraplegic subject, a biomechanical model, adapted from Riener and Fuhr [4], was implemented in Matlab Simulink^® ^(MathWorks, Inc. Massachusetts). The Plant was constrained to move in the sagittal plane and the knee was assumed to be an ideal hinge joint. The movement considered was the flexion extension of the knee. Inputs to the Plant were the pulse width of the stimuli delivered to the quadriceps through surface electrodes. The Plant output was the knee joint angle. Five muscle groups were considered: hamstrings (i.e. semimembranosus, semitendinosus, biceps femoris long head), bicep femoris short head, rectus femoris, vasti muscles, lateral and medial gastrocnemius.

Muscle groups could be treated independently and were characterized by activation and contraction parameters. Muscular activation included the effect of spatial summation (through the recruitment curve), the effect of temporal summation (through the calcium dynamics) and the muscular fatigue. When the quadriceps were stimulated with a pulse width greater than the recruitment threshold (100 μs), other muscles still contributed to limb dynamics by their passive viscous and elastic properties. The dynamic modellization took the elastic and the viscous torque into account (for more details see [4]).

To describe the effect of fatigue/recovery, a fitness function *fit*(*t*) was used [4]. It can be expressed by the following first order relation:

dfit(t)dt=(fitmin⁡−fit(t))a(t)λ(f)Tfat+(1−fit(t))(1−a(t)λ(f))Trec     (eq. 1)
 MathType@MTEF@5@5@+=feaafiart1ev1aaatCvAUfKttLearuWrP9MDH5MBPbIqV92AaeXatLxBI9gBaebbnrfifHhDYfgasaacH8akY=wiFfYdH8Gipec8Eeeu0xXdbba9frFj0=OqFfea0dXdd9vqai=hGuQ8kuc9pgc9s8qqaq=dirpe0xb9q8qiLsFr0=vr0=vr0dc8meaabaqaciaacaGaaeqabaqabeGadaaakeaadaWcaaqaaiabdsgaKjabdAgaMjabdMgaPjabdsha0naabmaabaGaemiDaqhacaGLOaGaayzkaaaabaGaemizaqMaemiDaqhaaiabg2da9maalaaabaWaaeWaaeaacqWGMbGzcqWGPbqAcqWG0baDdaWgaaWcbaGagiyBa0MaeiyAaKMaeiOBa4gabeaakiabgkHiTiabdAgaMjabdMgaPjabdsha0naabmaabaGaemiDaqhacaGLOaGaayzkaaaacaGLOaGaayzkaaGaemyyae2aaeWaaeaacqWG0baDaiaawIcacaGLPaaaiiGacqWF7oaBdaqadaqaaiabdAgaMbGaayjkaiaawMcaaaqaaiabdsfaunaaBaaaleaacqWGMbGzcqWGHbqycqWG0baDaeqaaaaakiabgUcaRmaalaaabaWaaeWaaeaacqaIXaqmcqGHsislcqWGMbGzcqWGPbqAcqWG0baDdaqadaqaaiabdsha0bGaayjkaiaawMcaaaGaayjkaiaawMcaamaabmaabaGaeGymaeJaeyOeI0Iaemyyae2aaeWaaeaacqWG0baDaiaawIcacaGLPaaacqWF7oaBdaqadaqaaiabdAgaMbGaayjkaiaawMcaaaGaayjkaiaawMcaaaqaaiabdsfaunaaBaaaleaacqWGYbGCcqWGLbqzcqWGJbWyaeqaaaaakiaaxMaacaWLjaWaaeWaaeaacqqGLbqzcqqGXbqCcqGGUaGlcqqGGaaicqaIXaqmaiaawIcacaGLPaaaaaa@7EA3@

where *a*(*t*) was the activation of the not fatigued muscle and *fit*_*min *_was the minimum fitness parameter. The time constants for fatigue (*T*_*fat*_) and for recovery (*T*_*rec*_), as well as *fit*_*min*_, were estimated from stimulation experiments [4].

The term λ(*f*) was introduced by Riener and Fuhr [4] to better account for the fact that muscle fatigue rate strongly depends on stimulation frequency and it was expressed by the following relation:

λ(f)=1−β+β(f100)2for f<100 Hz     (eq. 2)
 MathType@MTEF@5@5@+=feaafiart1ev1aaatCvAUfKttLearuWrP9MDH5MBPbIqV92AaeXatLxBI9gBaebbnrfifHhDYfgasaacH8akY=wiFfYdH8Gipec8Eeeu0xXdbba9frFj0=OqFfea0dXdd9vqai=hGuQ8kuc9pgc9s8qqaq=dirpe0xb9q8qiLsFr0=vr0=vr0dc8meaabaqaciaacaGaaeqabaqabeGadaaakeaafaqabeqacaaabaacciGae83UdW2aaeWaaeaacqWGMbGzaiaawIcacaGLPaaacqGH9aqpcqaIXaqmcqGHsislcqWFYoGycqGHRaWkcqWFYoGydaqadaqaamaalaaabaGaemOzaygabaGaeGymaeJaeGimaaJaeGimaadaaaGaayjkaiaawMcaamaaCaaaleqabaGaeGOmaidaaaGcbaGaeeOzayMaee4Ba8MaeeOCaiNaeeiiaaIaemOzayMaeyipaWJaeGymaeJaeGimaaJaeGimaaJaeeiiaaIaeeisaGKaeeOEaOhaaiaaxMaacaWLjaWaaeWaaeaacqqGLbqzcqqGXbqCcqGGUaGlcqqGGaaicqaIYaGmaiaawIcacaGLPaaaaaa@54BE@

In our stimulations the stimulation frequency *f *was always fixed at 40 Hz and β was a shape factor not dependent on frequency or muscles.

Finally, the activation of the tiring muscle was given by:

*a*_*fat*_(*t*) = *a*(*t*)* *fit*(*t*)     (eq. 3)

The fatigue occurrence showed a decrease of the muscle input gain to 50% of its nominal value over 100 s, comparable to [17].

### Artificial Neural Network Inverse Model

Following direct-inverse modelling approach [22], the pulse width waveforms, used as ANNIM desired outputs, were rectified sinusoids and triangles of different duration and amplitude. The ANNIM inputs were obtained stimulating the nominal Plant, i.e., not including the fatigue effects (*fit*(*t*) = 1), in response to the chosen pulse width signals. In order to take the system dynamics into account, ANNIM inputs were augmented with signals corresponding to past inputs. Therefore, ANNIM inputs were the actual knee angle and velocity and their 4 previous samples (q(t), q(t-1), ..., q(t-4)) and (q˙
 MathType@MTEF@5@5@+=feaafiart1ev1aaatCvAUfKttLearuWrP9MDH5MBPbIqV92AaeXatLxBI9gBaebbnrfifHhDYfgasaacH8akY=wiFfYdH8Gipec8Eeeu0xXdbba9frFj0=OqFfea0dXdd9vqai=hGuQ8kuc9pgc9s8qqaq=dirpe0xb9q8qiLsFr0=vr0=vr0dc8meaabaqaciaacaGaaeqabaqabeGadaaakeaacuWGXbqCgaGaaaaa@2E20@(*t*), q˙
 MathType@MTEF@5@5@+=feaafiart1ev1aaatCvAUfKttLearuWrP9MDH5MBPbIqV92AaeXatLxBI9gBaebbnrfifHhDYfgasaacH8akY=wiFfYdH8Gipec8Eeeu0xXdbba9frFj0=OqFfea0dXdd9vqai=hGuQ8kuc9pgc9s8qqaq=dirpe0xb9q8qiLsFr0=vr0=vr0dc8meaabaqaciaacaGaaeqabaqabeGadaaakeaacuWGXbqCgaGaaaaa@2E20@(*t *- 1), ..., q˙
 MathType@MTEF@5@5@+=feaafiart1ev1aaatCvAUfKttLearuWrP9MDH5MBPbIqV92AaeXatLxBI9gBaebbnrfifHhDYfgasaacH8akY=wiFfYdH8Gipec8Eeeu0xXdbba9frFj0=OqFfea0dXdd9vqai=hGuQ8kuc9pgc9s8qqaq=dirpe0xb9q8qiLsFr0=vr0=vr0dc8meaabaqaciaacaGaaeqabaqabeGadaaakeaacuWGXbqCgaGaaaaa@2E20@(*t *- 4)). It has already been established that adding noise to the training data in artificial neural learning improves the quality of learning, as measured by the trained networks ability to maximize exploration of the input/output space, avoid overfitting and generalise [23]. Therefore, a white noise was added to the input signals (mean 0, standard deviation equal to 5% of the maximum pulse width value). Several networks were trained and the smallest network architecture that gave good RMSE and similar performance between training and testing data was chosen, as reported in details in a previous article of the authors [21]. The ANNIM was a multilayer FF perceptron with 10 input neurons, 10 neurons in hidden layer and 1 neuron in the output layer. We chose the hyperbolic tangent as the activation function of the hidden layer and the logarithmic sigmoid function in the output layer, mapping the non linearity of the Plant and the bounded stimulation range. The Levenberg-Marquardt learning algorithm was used to train ANNIM [24].

### Neuro feedback

**Figure 2:**
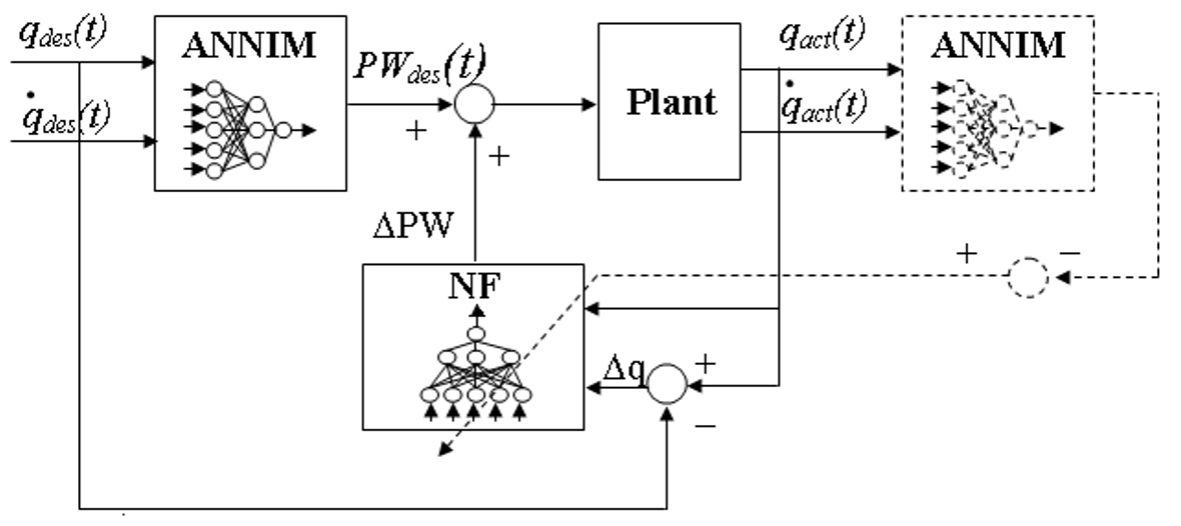
**NF Training scheme**. Scheme used to collect the training set of NF.

NF training set was obtained using a setup including the series of ANNIM, Plant and another ANNIM (Figure 2). This scheme was aimed at obtaining the relationship between the angular error and the pulse width signal during a repeated movement sequence, where the effect of muscular fatigue, as well as any time variant occurrence, was evident. Desired angle (q_des_) was input to the ANNIM, that had already been trained, producing the corresponding desired pulse width (PW_des_) as an output. PW_des _was then given as an input to the Plant, where fatigue was modelled. Output of the Plant was the actual angle (q_act_), i.e., the angle generated stimulating a Plant in which the fatigue effect was included. After that, q_act _was used as an input to the second ANNIM, which was exactly a copy of the first one, converting it in the PW domain producing PW_act_. PW_act _was the nominal pulse width corresponding to the actual movement q_act_. Therefore, the angular error Δ*q *= *q*_*act *_- *q*_*des *_was correlated to an estimation of the current fatigue level expressed in the pulse width domain: Δ*PW *= *PW*_*act *_- *PW*_*des*_.

These two signals were used as input/output couples for NF training set. Thus NF was trained to produce ΔPW as an output, when it received as an input the correspondent angular error Δq. This training set allowed NF to work as a predictor and a compensator of the fatigue effect: when the Plant was getting tired, the angular error (Δq) increased and NF gave an extra pulse width (ΔPW). Once trained NF allowed estimating the fatigue level and mapping the actual angular error into a needed correction in the pulse width domain.

**Figure 3:**
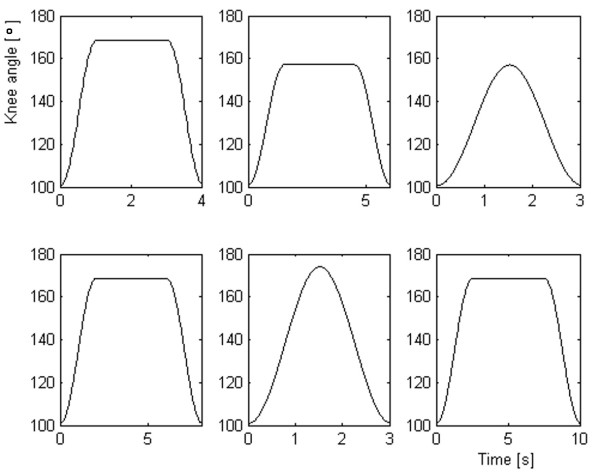
**Examples of the NF training signals**. Some examples of the first 10 seconds of the signals used to build the NF training set are reported in this figure. Each trajectory was delivered for 100 s to the setup reported in Figure 2 in order to obtain the Δq and ΔPW signals.

The signal used to build the training set of NF (q_des _in Figure 2) was a repeated sequence of consecutive flexion extension trajectories lasting 100 s. The training set included 12 angular trajectories lasting 100 s, having different profiles, durations and amplitudes; some examples of the first angular oscillation are reported in Figure 3.

The NF was a non-adaptive multilayered perceptron with 10 input neurons, 8 neurons in the hidden layer and 1 in the output layer. We chose the hyperbolic tangent as the activation function in order to allow positive and negative corrections of pulse width. The introduction of past inputs allowed the network to map the dynamic nature of the system. The training algorithm was Levenberg-Marquardt [24].

### Capability to resist to mechanical disturbances

More than the tracking performance and the capability to manage fatigue occurrence, the EMC controller proved its resistance to internal disturbances that could occur during the stimulation. Such disturbances could be caused by internal spastic muscle contractions or external loading of the limb. In order to model a mechanical disturbance such as a spasm, a square wave lasting for two seconds was delivered to the simulator with the limb in different positions during the simulated movement. The spasm amplitude ranged between 20% and 30% of the maximal total torque of the knee: the spasm model was analogous to [17].

An additional test on the effect of a distributed noise on the knee torque was designed to check the capability of the controller to face random variations in the Plant. This test could simulate an error in the stimulation or in the electrodes coupling with the skin. Random noises uniformly distributed between ± 25%, ± 30%, ± 35%, ± 40%, ± 45% and ± 50% of the maximal knee torque were tested, as in Abbas et al. [17].

### EMC robustness

EMC capabilities to track time varying physical parameters, indicating an increase or a decrease of the fitness level of the subject, were tested as a second aspect of this methodological study. In particular, the robustness of our controller was tested changing the following parameters: the damping property of the leg, the time constant of fatigue and recovery and the weight of the limb. The values of these coefficients were fixed "a priori" in the model. For this reason, the training of the ANNs of EMC was not including any variation of such parameters. Anyway, ANNs generalization capability could partly adapt to these possible variations.

All these parameters were changed up to ± 50% of their nominal value and the angular RMSE on the 1^st ^(not fatigued) and the 5^th ^(fatigued) flexion extension of a repetitive trial were assessed.

### Reference controllers anti wind-up PID (PIDAW) and NEUROPID

In order to prove advantages of EMC strategy, a comparison with two reference controllers was performed: a traditional closed loop controller PID and the model-based neural controller, NEUROPID, proposed by Chang [16].

The PID controller general form in the time domain is given by:

u(t)=KP(t)+Ki∫0te(τ)dτ+Kdde(t)dt
MathType@MTEF@5@5@+=feaafiart1ev1aaatCvAUfKttLearuWrP9MDH5MBPbIqV92AaeXatLxBI9gBaebbnrfifHhDYfgasaacH8akY=wiFfYdH8Gipec8Eeeu0xXdbba9frFj0=OqFfea0dXdd9vqai=hGuQ8kuc9pgc9s8qqaq=dirpe0xb9q8qiLsFr0=vr0=vr0dc8meaabaqaciaacaGaaeqabaqabeGadaaakeaacqWG1bqDcqGGOaakcqWG0baDcqGGPaqkcqGH9aqpcqWGlbWsdaWgaaWcbaGaemiuaafabeaakiabcIcaOiabdsha0jabcMcaPiabgUcaRiabdUealnaaBaaaleaacqWGPbqAaeqaaOWaa8qCaeaacqWGLbqzcqGGOaakiiGacqWFepaDcqGGPaqkcqWGKbazcqWFepaDcqGHRaWkcqWGlbWsdaWgaaWcbaGaemizaqgabeaakmaalaaabaGaemizaqMaemyzauMaeiikaGIaemiDaqNaeiykaKcabaGaemizaqMaemiDaqhaaaWcbaGaeGimaadabaGaemiDaqhaniabgUIiYdaaaa@5446@

where: *e*(*t*) is the difference between the reference and the actual value of the controlled variable, and *Kp*, *Ki*, *Kd*, are the proportional, integrative and derivative parameters respectively.

The PID controller parameters were first identified using an iterative procedure based on the minimization of Root Mean Square Error (RMSE) [25], where the initial estimation of the optimization was derived from the Ziegler-Nichols rules [26]. Then the transfer function of the PID was discretized in view of a digital implementation of the control algorithm.

A saturation block was added between the output of the PID controller and the stimulator input in order to limit the pulse width values between 0 and 500 μs. The use of integral action in the PID controller combined with actuator saturation can give undesirable effects: if the error signal is so large that the integrator saturates the actuator, the feedback path will be broken because the actuator would remain saturated even if the Plant output changed. The integrator, being an unstable system, may then integrate up to large values. When the error is finally reduced, the integration may be so large that it will take much time before the output of the integrator falls to a normal value. This effect is called integrator wind-up. To avoid it, a PID was introduced in an anti wind-up scheme [27], in the following PIDAW.

The NEUROPID controller, developed by Chang at al. [16], included an ANN in the FF loop, which was the inverse model of the system, and a PID in the feedback loop, which was able to adjust the pulse width signal in case of error between the desired and the actual angle.

In order to compare the three listed controllers (PIDAW, NEUROPID, EMC), we simulated controlled repeating sequences of flexion extension movements lasting 100 s and we computed the RMSE between actual and desired angular values.

A non parametric Kruskal-Wallis test (p < 0.05) was carried out to highlight significant differences between the RMSE obtained by the three controllers at different levels of fatigue. A Dunn-Sidak post hoc test was performed to understand which pairs of effects were significantly different.

## Results

### Tracking performance

**Figure 4:**
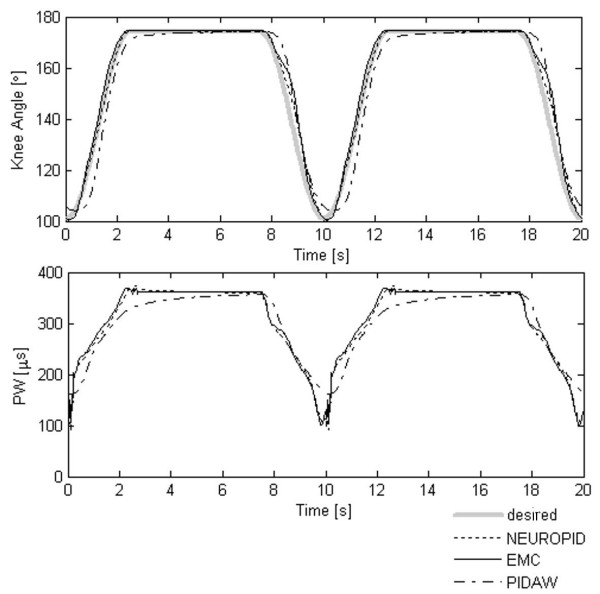
**EMC vs traditional controllers without fatigue**. A comparison of the performance obtained by the three controllers in term of angular trajectories and pulse width without considering the muscular fatigue effect.

In Figure 4 the tracking performance of the three controllers (EMC, PIDAW and NEUROPID) is shown in the case of no fatigue.

Without fatigue, the tracking capability of EMC was very similar to the NEUROPID one, while the PIDAW showed the typical time lag. The RMSE between the desired and actual trajectory shown in Figure 4 was about 1,7° for EMC, 7,7° for the PIDAW and 3.2° for NEUROPID.

### Fatigue mapping

**Figure 5:**
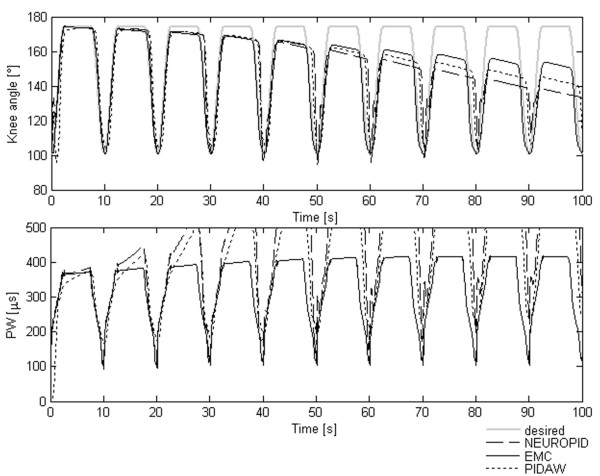
**EMC vs traditional controllers with fatigue**. An example of the comparison of the performance obtained by the three controllers in terms of angular trajectories and pulse width. The testing signal lasted 100 s and during the trial fatigue was strongly affecting the Plant performance.

In order to test fatigue mapping capabilities, the comparison of the three controllers was performed in terms of the RMSE obtained in response to simulations of 100 s using 6 different angular trajectories (repeated oscillations of different amplitudes, from 40 to 70 degrees and each oscillation lasted from 2 to 10 seconds). In Figure 5 an example of the performance of the three controllers with fatigue is reported. In this case, the three controllers behaved very differently: PIDAW and NEUROPID increased the stimulation pulse width rapidly, due to the increasing tracking error. Within the third cycle, the pulse width raised up to the limit (500 μs) and in the next repetition it remained saturated for more time. Unfortunately, due to fatigue, such stimulation did not achieve the correct tracking of the desired path and it was greatly tiring out the Plant. In addition, in between two successive cycles, those two controllers were not suspending the stimulation but they only reduced it. The continuous stimulation did not permit the possibility of recovery. In contrast, the EMC was always able to keep the stimulation at lower levels. In this way, the fatigue was increasing more slowly and the exercise was repeated with more amplitude for much longer. The EMC avoided over-stimulating the Plant in reaching the desired trajectory when fatigue was too strong and it always had an interval of no stimulation in between waves, which was fundamental for recovery. In this way, it was able to prolong the exercise with satisfying extensions.

The three controllers were tested in response to different testing signals lasting 100 s not included in the training set of both the ANNs of the EMC. Between 90 and 100 s the mean value of the RMSE with respect to the desired knee angle trajectories tested was about 14° for the EMC, while it was about 21° for the PIDAW and 23° for the NEUROPID.

**Figure 6:**
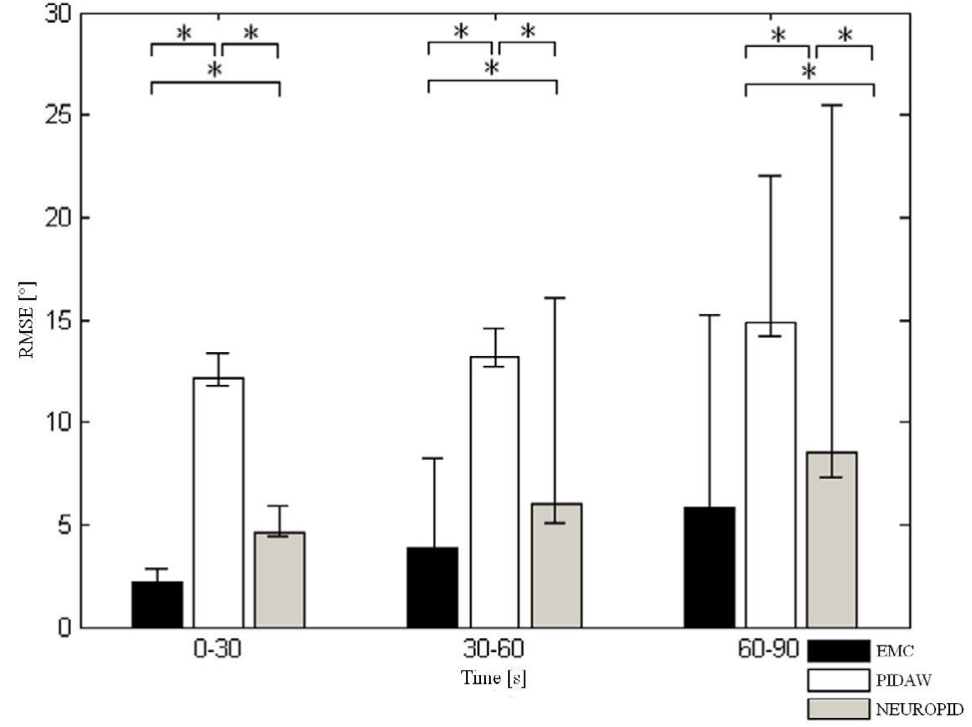
**Statistical comparison of EMC vs traditional controllers**. Comparison of the performance obtained by EMC, PIDAW and NEUROPID in terms of the median and the quartiles of the RMSE obtained on 6 different testing angular trajectories. Such comparison was divided in three periods (0–30 s, 30–60 s and 60–90 s). The Kruskal-Wallis test highlighted significant differences between the controllers. The asterisks indicate that the Dunn-Sidak post-hoc test showed a significant difference between the RMSE.

The results of the Kruskal Wallis test is reported in Figure 6 and highlighted that there were significant statistical differences between the RMSE obtained by the three controllers in three different periods of time (0–30 s, 30–60 s, 60–90 s). The Dunn-Sidak post hoc test showed that a significant difference was present between all the controllers in all the time periods.

### Resistance to disturbances

**Figure 7:**
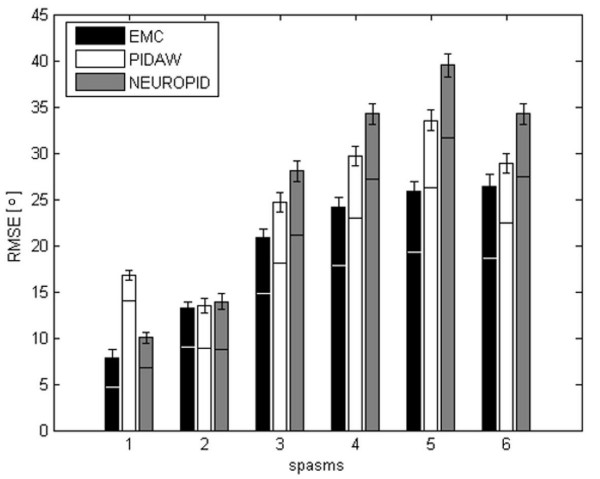
**Capability to react to spasms**. Comparison of the three controllers (EMC, PIDAW and NEUROPID) performed in terms of the RMSE during flexion extension lasting 100 s. X axis represents the events indicating spasms occurrence during the movement. 6 spasms were randomly added to the 100 s angular trajectories. Each spasm lasted 2 s and its amplitude was varied from 20% and 30% of the maximal total torque of the knee.

In order to test resistance to internal mechanical disturbances (like occurring spasms), the comparison of the three controllers was performed in terms of the RMSE during flexion extension movements lasting 100 s. Six spasms occurrences, each lasting 2 s, were randomly added to the Plant knee torque during the 100 s simulation, both during the extension and flexion. For each spasm, different amplitudes were tested (between 20% and 30% of the maximal total torque of the knee). Performances of the three controllers are reported in Figure 7. The increase of the RMSE due to the spasms, evidenced by the lines crossing each column in Figure 7, was very similar for the three controllers in the early spasms as well as in the later ones, independently from the phase of the cycle. These results demonstrated that even if the EMC was never trained to respond to such disturbances both the stability of the system and its capability to generalize to unknown events was comparable to the other two reference controllers, keeping anyway the specific advantages on fatigue estimation.

**Figure 8:**
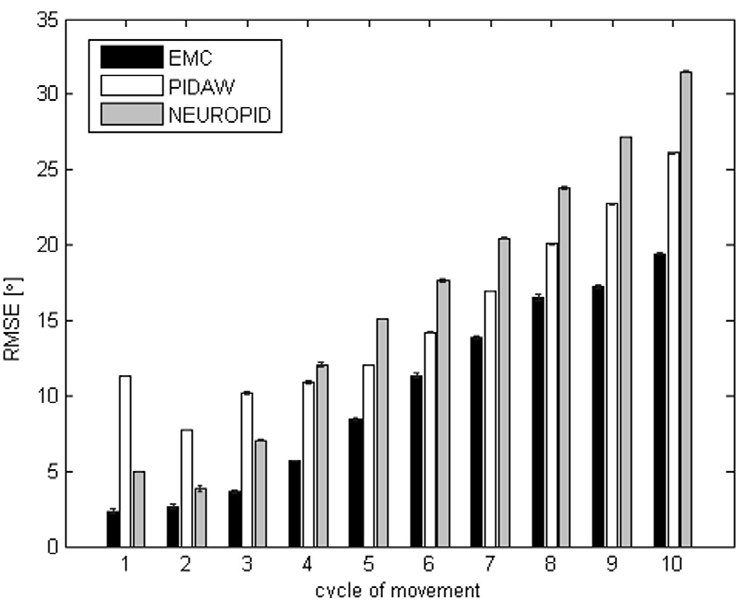
**Capability to react to distributed noise**. Comparison of the three controllers performed in terms of the RMSE during flexion extension sequence of 10 oscillations for a total duration of 100 s. Random noises of different amplitudes were added on the knee torque (ranging from 25% to 50% of the maximum knee torque values). The RMSE was evaluated separately for each oscillation of the angular trajectory (X axis), hence at different levels of fatigue.

In order to test resistance to random noises of different amplitudes (ranging from 25% to 50% of the knee torque), the comparison of the three controllers was performed in terms of the RMSE during flexion extension movements lasting 100 s. A random noise was added to the whole sequence. The EMC had the best performance reducing evidently fatigue effect and tracking discrepancy, both in the initial oscillations (without fatigue) and for the last oscillations (9–10^th^) when fatigue is strongly affecting the Plant performances (Figure 8).

### Robustness

**Figure 9:**
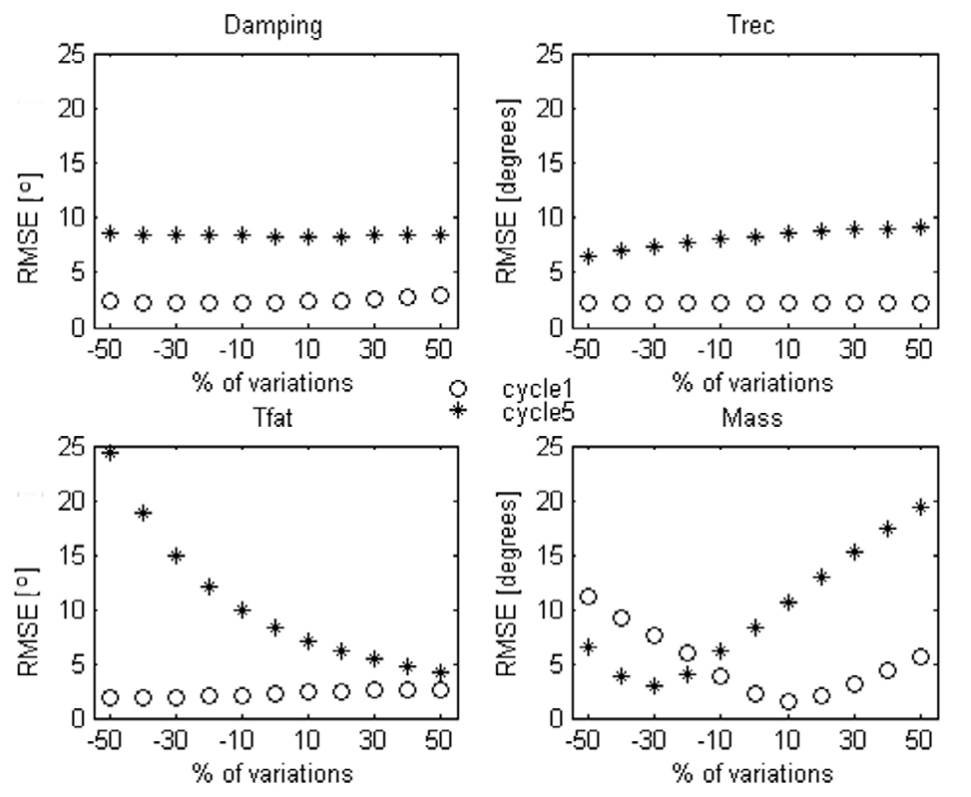
**EMC robustness**. EMC generalization performance obtained changing 4 parameters: damping, T_rec_, T_fat _and the limb mass. The horizontal axis indicates the percent by which the parameter has been varied, the vertical axis indicates the RMSE error. The errors in the first oscillation (wave 1, reported in circles) and in the fifth (wave 5 reported in asterisks) of a repetitive angular trajectory are shown.

EMC robustness with respect to changes in the Plant parameters was tested by calculating the error in tracking performance and the results are shown in Figure 9. The circles represent the error on the first flexion extension (wave1), while asterisks represent the values of the RMSE on the fifth flexion extension, i.e., after about 50 s of stimulation (wave 5).

Modifications in the viscoelastic properties, i.e., damping value, of the Plant were compensated very well by the EMC, damping changes of 50% affected the results less than 1° both in the first and in the fifth leg movement. Analogously, the EMC coped with the changes in the time required for recovery from fatigue, (T_rec_), well. As expected a slight increase of the RMSE was obtained when T_rec _was increased. Naturally, the first wave was not affected much by the variation of this parameter, like the variation in T_fat_, because fatigue was not yet present at this stage of the movement. The effect of variations of T_fat _was much more evident, when negative variations of T_fat _were simulated (meaning a faster occurrence of fatigue) and in fact the RMSE increases exponentially in the left part of the panel referred to as T_fat _in Figure 9. On the contrary, positive variations of T_fat _reduced the error. Indeed, the EMC was trained to face fatigue up to the defined value of T_fat_; higher values of T_fat _indicated a slower fatiguing, well addressed by the EMC. Lower values of T_fat_, on the contrary, were not in anyway included in the training set. The robustness of the controller when lower leg mass was simulated, was good in the case of a reduction of the mass. In this case, while an overshooting was shown at the first cycle, once the error was detected by the feedback, NF correction reduced the error (asterisks lower than circles). On the contrary, in case of an increase of the mass of the leg, the effect was very similar to when fatigue occurred faster, showing a quick increase of the error. However, positive variations of 20% led to error of less than 10°.

## Discussion

The EMC showed good tracking performance when fatigue phenomenon was not present or stayed at low levels. In those cases, the EMC was more accurate with respect to the other two controllers tested, especially in avoiding the PID time lag. Similar levels of angular errors were showed by other controllers proposed in literature, like the Sliding Mode Controller [20]. Namely, the EMC tracking error on the same trajectories used by Jezernik was about 4.5°, which is quite comparable to the best result reported by those authors (about 3°).

However, the most significant advantage of the EMC was visible when fatigue was great. The behaviour of the EMC during the process of tiring was completely different to the other two controllers, PIDAW and NEUROPID, reducing the RMSE by a third after 100 s.

The EMC achieved such different performance because the NF correction considered tracking of the desired trajectory as well as the level of fatigue. The training solution of the EMC translated the angular error into pulse width correction estimating the differences between the actual fatigued performance with respect to the nominal one. In this way, the EMC corrected the stimulation parameters by giving an extra pulse width correlated to the level of fatigue. The main effect of this strategy was that stimulation parameters grew much more slowly during repeated flexion extensions, thereby not saturating and not overstressing the stimulated muscle. This behaviour was exactly contrary to PID based controllers [1,15,16,21]. The latter stimulated the muscle to a maximum, depending only on the angular error and not evaluating the feasibility of tracking. This solution, once fatigue was too strong to permit proper tracking, caused an over-stimulation of the muscle, inducing an even more rapid fatigue ramp. Analogously, the PG/PS controller proposed by Reiss and Abbas [19] had the same philosophy of the PID, being the adaptive controller tuned by a PD controller on the angular error only. Anyway, not a complete test on fatigue managing was available for the PG/PS controller, being fatigue included in the muscle model only in the simulations discussed in [17], where the testing trajectory was very small in amplitude (25°), lasted just 10 s, with a stimulation frequency of 20 Hz. Such testing trajectory is completely different from those used in EMC training and testing and, anyway, is not adequate to verify the capability of coping the fatigue occurrence as specifically aimed in the EMC design.

In addition, the EMC was able to resist well to mechanical disturbances, even if such occurrences were not included in the examples used for training. This property was similar to PID based controller, thereby maintaining the advantage of the best fatigue mapping learnt by the EMC.

Robustness in the model parameters was tested and the satisfactory results obtained ensured good generalization for successive sessions on the same subject, especially in the case of a good muscle conditioning. It has to be mentioned that offline, after each single session, depending on the observed errors, an extra training of NF could be performed if necessary.

To verify the stability of the EMC controller for step and ramp knee movements, analogously to Jezernik *et al*. [20] and the EMC remained always stable. Instability was never observed in all the experiments carried out in this study.

EMC training on the preparation of the exercise is a crucial point in the clinical applicability of the controller. Actually, in order to train the inverse model (ANNIM) the subject needs to be stimulated with a variety of pulse width shapes and the corresponding knee angle are recorded. The set of pulse width/angle forms the training set of the ANNIM when the task is done in nominal conditions (no fatigue) or in initial single movements with long rest phases in between. Once such network is trained, the subject is stimulated longer, inducing fatigue, in a following session. A set of trajectory errors will be used as training input of the NF neural network and the corresponding desired output will be built using the replication of the ANNIM (Figure 2). These ANN training sets could be collected during the conditioning period, when patients usually become familiar with electrical stimulation and increase muscular tone. In such a way, a conditioning period, which normally takes place before the controlled stimulation session, is exploited for controller training. By the way these collection procedures do not globally change the programme for patients and do not require any specific setup except the one which will be used for the functional neuroprosthesis, simplifying therapists' efforts. This aspect is one important advantage with respect to other controllers discussed in literature, such all those based on PID [17-19,25,26] as well as model based controllers [25] and sliding mode controllers [20].

The last point concerns the possibility to generalize the EMC control strategy to more complex motor tasks. In these experiments, we utilized a cyclic joint angle tracking task to evaluate the performance of the control system. This task, which has been used in the evaluation of several neuroprosthesis control systems in the past [1,15-21,25] may represent a simplified version of practical actions that could be performed with FES systems, such as: FES exercise systems that utilize cyclic movements and lower-extremity FES systems for generating patterned movements such as gait, side-stepping, and stair-climbing. More importantly, however, the task used in these experiments demonstrates the ability of the controller to automatically account for the subject-specific musculo-skeletal input/output properties, and for fatigue occurrence that would be exhibited in many FES tasks.

Supporting a good translational property of EMC over multiple muscles and more complex tasks two points should be considered: first, EMC do not use any extra setup to identify the parameters of the controller. Second, neural networks can process many inputs and have many outputs; they are readily applicable to multivariable systems.

## Conclusion

We proposed a controller, called EMC, for neuromuscular stimulation of knee flexion extension which is composed by a feedforward inverse model and a feedback controller, both implemented using neural networks. The training of the networks is conceived to avoid to a therapist and a patient any extra experiment, being the collection of the training set included in the normal conditioning exercises. The EMC philosophy differs from classical feedback controllers because it does not merely react to the error in the tracking of the desired trajectory, but it estimates also the actual level of fatigue of the muscles. This solution allows to prolong the exercise improving the conditioning effects. In addition, the controller robustness was tested, demonstrating a good capability of generalizing and thus reducing the time consuming for re-training, especially if subjects conditions are improving.

## Competing interests

The author(s) declare that they have no competing interests.

## Authors' contributions

AP and SF have made substantial contributions to conception and design, acquisition of data, analysis and interpretation of data and manuscript drafting; EDM have made part of acquisition of data, analysis and interpretation of data and have been involved in drafting the manuscript; and GF have made substantial contributions to conception and design and interpretation of data and have given final approval of the version to be published.
