# Correction: Error mapping controller: a closed loop neuroprosthesis controlled by artificial neural networks

**DOI:** 10.1186/1743-0003-4-9

**Published:** 2007-04-20

**Authors:** Alessandra Pedrocchi, Simona Ferrante, Elena De Momi, Giancarlo Ferrigno

**Affiliations:** 1 Nitlab, Bioengineering Department, Politecnico di Milano, Via Garofalo, 39, 20133, Milano, Italy

## 

After the publication of this work [1], we noticed in figure 2 (see Figure 1) the signal indicated as the desired output to train NF was not correctly reported in the figure. The training signal is the result of the difference between PW_act _and PW_des _as it is explained in the text, and as it is shown in the new figure we are showing here.

**Figure 1:**
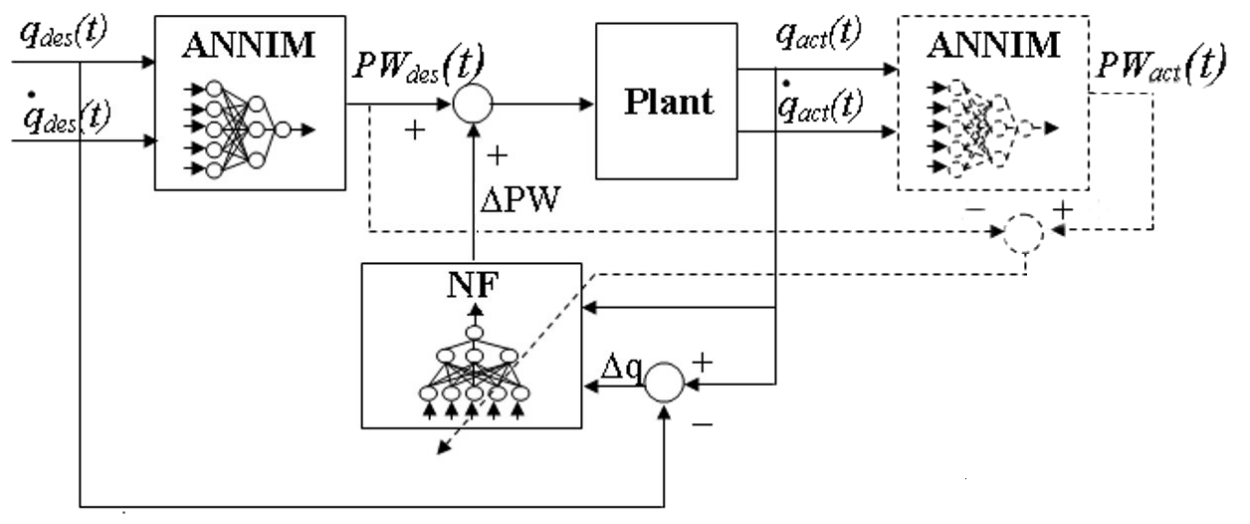
**NF Training scheme**. Scheme used to collect the training set of NF. The training output signal of NF is the difference between PW_act _and PW_des _and not the PW_act _as it was wrongly indicated in figure 2 of the original paper.

We apologize for the inconvenience that this inaccuracy in the paper might have caused to the readers.
